# Primary Ovarian Perivascular Epithelioid Cell Tumor Diagnosed During Pregnancy: A Case Report and Review of the Literature

**DOI:** 10.7759/cureus.91959

**Published:** 2025-09-10

**Authors:** Iqra Yasin, Afshan Saeed Usmani

**Affiliations:** 1 Gynecologic Oncology, Surgical Oncology, Shaukat Khanum Memorial Cancer Hospital and Research Centre, Lahore, PAK; 2 Obstetrics and Gynecology, Southend University Hospital, Southend-on-Sea, GBR

**Keywords:** benign and malignant, ovarian cancer surgery, pregnancy cancer, rare ovarian tumor, perivascular epithelioid cell tumor (pecoma)

## Abstract

Perivascular epithelioid cell tumors (PEComas) are rare mesenchymal neoplasms with dual melanocytic and smooth muscle differentiation. Ovarian PEComas are extremely rare, and to our knowledge, this is the first reported case diagnosed during pregnancy.

A 31-year-old pregnant woman underwent resection of a left adnexal mass at an external facility. Histopathology assessed at the pathology department of Shaukat Khanum Memorial Cancer Hospital & Research Centre (SKMCH&RC) revealed a PEComa. Follow-up imaging at our center showed a residual complex solid-cystic mass in the left adnexa. Multidisciplinary discussion led to fertility-sparing surgical staging, including left salpingo-oophorectomy, pelvic wall mass excision, and infracolic omentectomy. Histopathology confirmed primary ovarian PEComa with metastatic deposits in the pelvic sidewall and omentum. Targeted therapy was deferred until after delivery. Microscopically, the tumor displayed epithelioid and spindle cells with perivascular arrangement and eosinophilic cytoplasm. Immunohistochemistry was positive for human melanoma black-45 (HMB-45) and smooth muscle actin. Features of malignancy included necrosis, mitotic activity of more than two per 50 high-power field (HPF), and vascular invasion.

A review of 12 published cases of ovarian PEComa revealed variable morphology and outcomes. Malignant potential is best predicted by tumor size, mitotic index, necrosis, and nuclear atypia. While surgery remains the primary treatment, the role of adjuvant therapy is evolving. This case highlights the diagnostic challenge of ovarian PEComa during pregnancy and emphasizes the importance of histologic classification and multidisciplinary management in guiding treatment decisions.

## Introduction

Perivascular epithelioid cell tumors (PEComas) are rare mesenchymal neoplasms of perivascular epithelioid cell (PEC) lineage, characterized by dual immunophenotypic expression of melanocytic and smooth muscle markers [1]. These tumors lack a known normal histological counterpart [2]. Early reports suggested their origin from vessel walls or unusual muscle cells, while embryological and in vitro studies indicate a neural crest stem cell origin during embryogenesis or tissue repair processes [3].

Microscopically, PEComas consist of nests or sheets of epithelioid or spindle cells with minimal intervening stroma. These cells have clear or granular eosinophilic cytoplasm, centrally located round to oval nuclei with nucleoli, and demonstrate a characteristic perivascular distribution around small- to medium-sized vessels [4]. Morphological variants range from pure spindle to mixed or epithelioid forms, occasionally demonstrating rare features such as sex cord-like differentiation and prolactin secretion [5,6].

Bonetti et al. first coined the term “perivascular epithelioid” in 1992 to describe angiomyolipoma (AML) and clear cell “sugar” tumors (CCST) of the lung-lesions showing perivascular distribution and unique morphology/immunophenotype [7]. Zamboni et al. later described pancreatic tumors with resemblance to CCST, thereby formally introducing the PEC concept [8]. The World Health Organization (WHO) in 2002 classified PEComas as a family of mesenchymal tumors defined by unique histological and immunohistochemical features. These include perivascular epithelioid morphology and co-expression of myomelanocytic markers [9].

Malignant PEComas are exceedingly rare, with an estimated global incidence of 0.12-0.24 per million [1]. The PEComa spectrum includes AML, lymphangioleiomyomatosis (LAM), CCST of the lung, clear cell myomelanocytic tumor (CCMT) of the falciform ligament, and primary extrapulmonary sugar tumors [10]. These tumors primarily affect abdominopelvic organs, including the kidney, uterus, and gastrointestinal tract [11]. Approximately 25% of all PEComas involve the gynecologic tract, affecting the uterus (most commonly), cervix, vagina, vulva, adnexa, and broad ligament [12].

PEComas are associated with tuberous sclerosis complex (TSC), which is characterized by multisystem hamartomatous lesions, including the brain, kidney, and heart. Renal AML is the prototypical PEComa linked with TSC, followed by LAM. Mutations in TSC1 (9q34) and TSC2 (16q13), affecting hamartin and tuberin, disrupt mammalian target of rapamycin (mTOR) pathway regulation via the Rheb/mTOR/p70S6K axis, forming the basis for targeted therapy with mTOR inhibitors [13]. While 80% of TSC patients develop AML, less than 50% of renal AMLs and under 10% of extra-renal AMLs are associated with TSC [14]. TFE3 gene rearrangement, another pathogenic mechanism observed in TSC-wildtype PEComas, correlates with poor mTOR inhibitor response [15].

Three major classification systems proposed by Folpe et al., Schoolmeester et al., and Bennett et al. stratify gynecologic PEComas based on histologic features associated with malignant potential [4,16,17]. Folpe et al. classify tumors as benign when no worrisome features are present, as uncertain malignant potential when fewer than four features exist, and as malignant when four or more are present, with key features including size ≥5 cm, high mitotic rate (>1/50 HPF), necrosis, high nuclear grade, and vascular invasion [16]. Conlon et al. modified the original Folpe classification [3]. Schoolmeester et al. refined this by requiring at least four features to label a tumor malignant, reducing false positives and combining benign and uncertain tumors into one category [4]. Bennett et al. further simplified the model, designating tumors as malignant if two or more adverse features are present and eliminating the term “benign” altogether, grouping all others under “uncertain malignant potential” to reflect diagnostic uncertainty [17].

Immunohistochemically, PEComas express both melanocytic markers (human melanoma black-45 (HMB-45), Melan-A/Mart-1, tyrosinase, MiTF, HMSA-1) and smooth muscle/myogenic markers (smooth muscle actin (SMA), calponin, h-caldesmon, and desmin) [12]. Additional markers such as S-100, CD34, EMA, PAX8, TFE3, and cathepsin K may also be expressed. Cathepsin K, though sensitive, lacks specificity, being seen in melanoma and alveolar soft part sarcoma [4]. Marker expression varies with morphology: spindle cell forms favor muscle markers, while epithelioid forms exhibit stronger melanocytic staining [3].

Differential diagnoses include clear cell tumors such as gastrointestinal stromal tumors (GISTs), melanoma, clear cell sarcoma, smooth muscle tumors, adrenal cortical carcinoma, rhabdomyosarcoma, myoepithelial tumors, and alveolar soft part sarcoma [18].

There is no standardized treatment protocol for PEComas. Surgery remains the mainstay, even in the presence of metastasis. Preoperative diagnosis is uncommon, making surgical intervention both diagnostic and therapeutic. Chemotherapy shows mixed results, with no optimal regimen established. Radiotherapy plays a limited role. PEComas may recur or metastasize, particularly to the lungs, even decades post-resection. mTOR inhibitors represent a promising targeted therapy in select molecular subtypes [1]. To date, a total of twelve primary ovarian PEComas have been reported in the English literature. These include two benign PEComas: epithelioid AML [19] and rhabdoid myomelanocytic tumor [20]; two PEComas of uncertain malignant potential [4,15]; and eight malignant PEComas, including malignant sclerosing PEComa [21] and malignant PEComa not otherwise specified (NOS) [4,18,22-26]. To our knowledge, this is the first reported case of primary ovarian malignant PEComa diagnosed during pregnancy, contributing to the limited literature and highlighting successful management with fertility-sparing surgical staging followed by planned deferred targeted therapy.

This case report was previously presented as an e-poster presentation at the Royal College of Obstetricians & Gynaecologists (RCOG) World Congress 2025, held from June 23 to 25, 2025, at ExCeL London.

## Case presentation

A 31-year-old pregnant female, in her second trimester, with two prior miscarriages and no known comorbidities, underwent suboptimal surgical excision of a left adnexal mass at an external institution after she complained of lower abdominal pain. Histopathological evaluation done at Shaukat Khanum Memorial Cancer Hospital & Research Centre (SKMCH&RC) confirmed the diagnosis of a PEComa.

**Figure 1:**
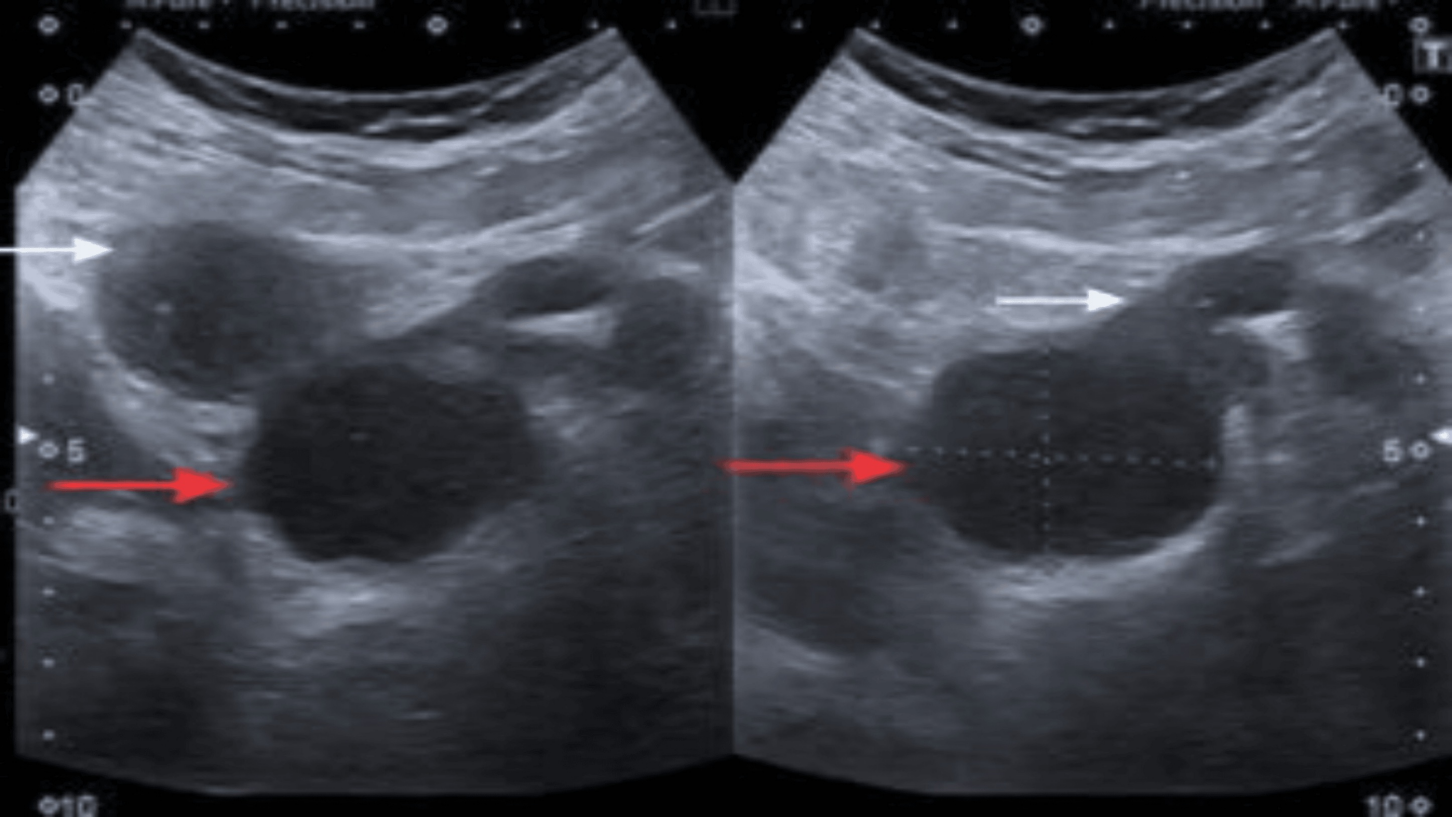
Transabdominal grayscale ultrasound demonstrating a large, complex left adnexal mass (cystic component indicated by red arrow and solid component indicated by white arrow). The lesion is predominantly cystic with internal septations and contains a solid, hypoechoic component. The findings are suspicious for malignancy and prompted further evaluation with MRI.

**Figure 2:**
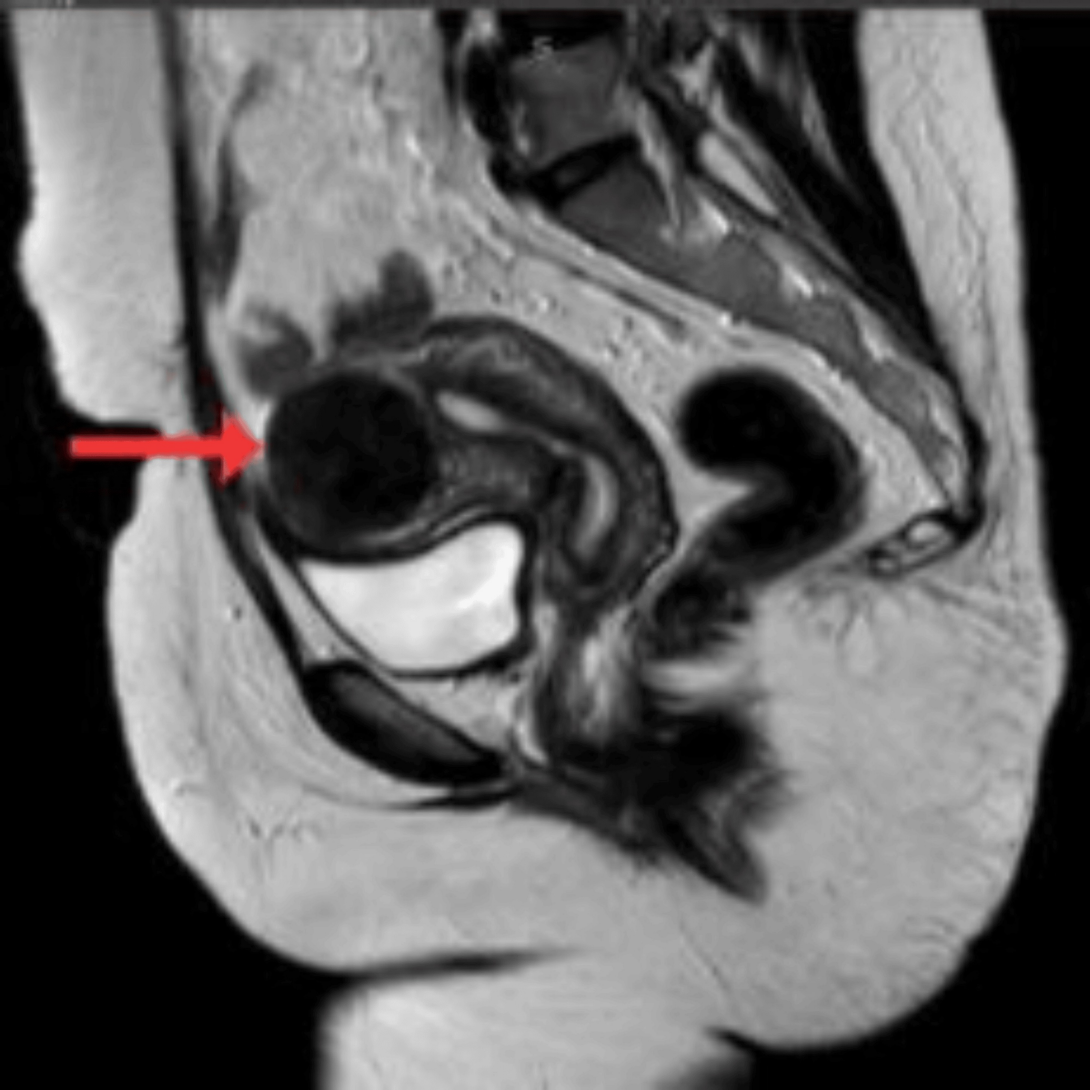
Sagittal T2-weighted MRI image of the pelvis showing a large, well-circumscribed heterogeneous mass (red arrow) with both cystic and solid components, arising from the left adnexal region. The mass demonstrates a hyperintense signal on T2, suggestive of fluid content, and displaces adjacent pelvic structures without clear evidence of infiltration, supporting a diagnosis of a potentially malignant ovarian lesion.

**Figure 3:**
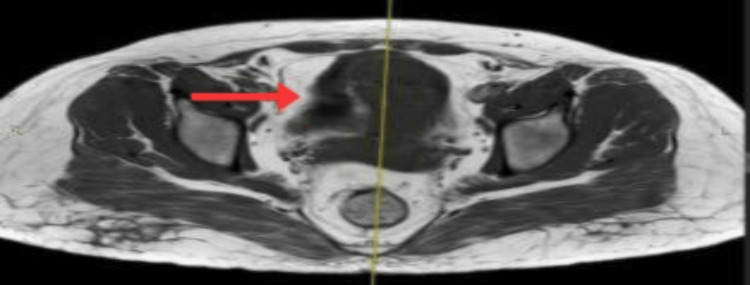
Axial T2-weighted MRI of the pelvis showing a complex left adnexal mass (red arrow) with cystic hyperintense and solid intermediate-signal components. The mass shows both solid and cystic characteristics, with hyperintense fluid content, further indicating a complex ovarian tumor consistent with perivascular epithelioid cell tumors (PEComa).

Follow-up pelvic ultrasound (Figure 1), followed by cross-sectional imaging of the pelvis, MRI (Figures 2, 3), done at our institution, revealed a residual complex solid-cystic mass in the left adnexa, with interval regression in both the solid and cystic components. Multidisciplinary team (MDT) discussion at SKMCH recommended fertility-sparing surgical staging.

**Figure 4:**
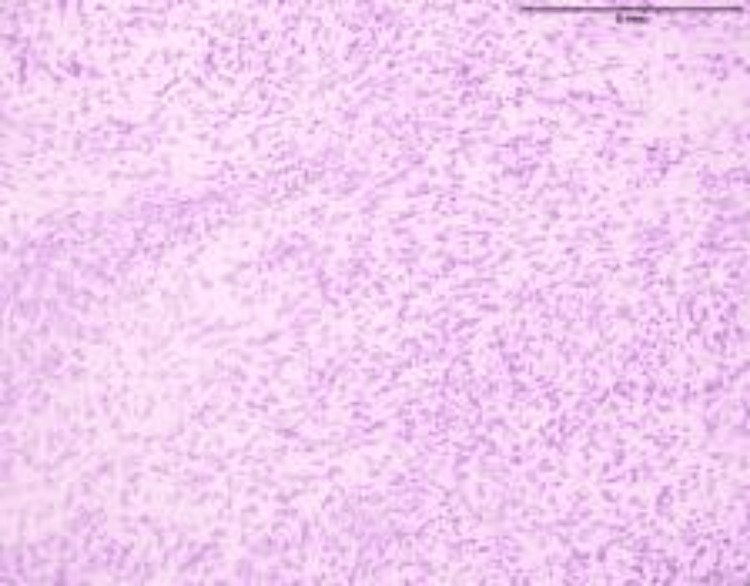
Ovarian perivascular epithelioid cell tumor (H&E stain, ×100 magnification). The low-power photomicrograph reveals a well-circumscribed tumor composed of nests and fascicles of epithelioid to spindle-shaped cells arranged around a prominent vascular network. The cells have clear to lightly eosinophilic cytoplasm and uniform nuclei with fine chromatin. No necrosis is seen in this field. On further immunohistochemistry, tumor cells demonstrated strong cytoplasmic positivity for human melanoma black 45 (HMB-45), confirming melanocytic differentiation.

**Figure 5:**
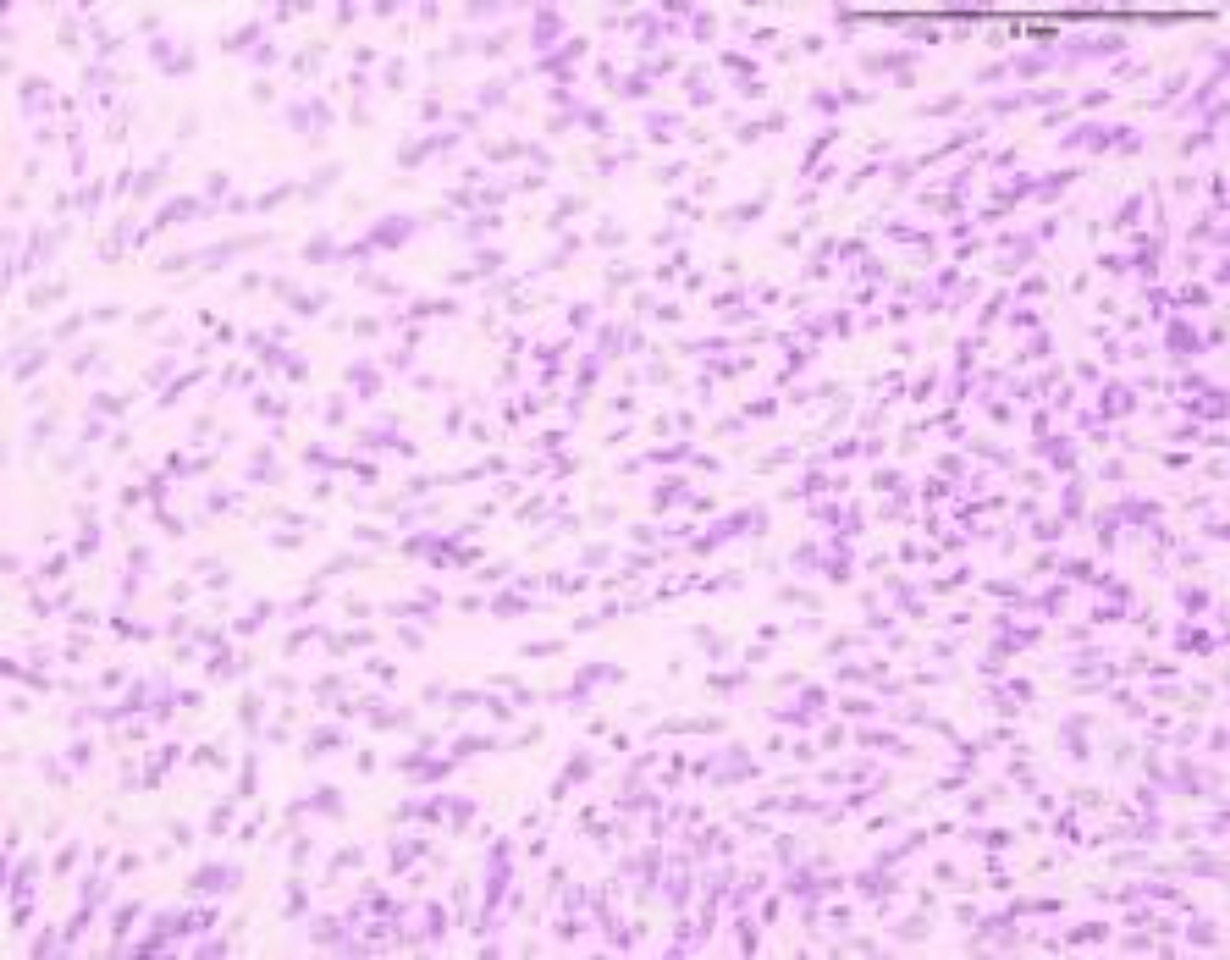
Ovarian perivascular epithelioid cell tumor (H&E stain, ×200 magnification). High-power view revealing epithelioid to spindle-shaped tumor cells with elongated nuclei, fine chromatin, and moderate amounts of eosinophilic cytoplasm. The cells are arranged in fascicles with a delicate vascular network. No significant nuclear pleomorphism or high mitotic activity is noted in this field. Immunohistochemistry on the same tumor demonstrated strong cytoplasmic human melanoma black 45 (HMB-45) positivity, confirming the melanocytic differentiation of ovarian perivascular epithelioid cell tumor (PEComa).

**Figure 6:**
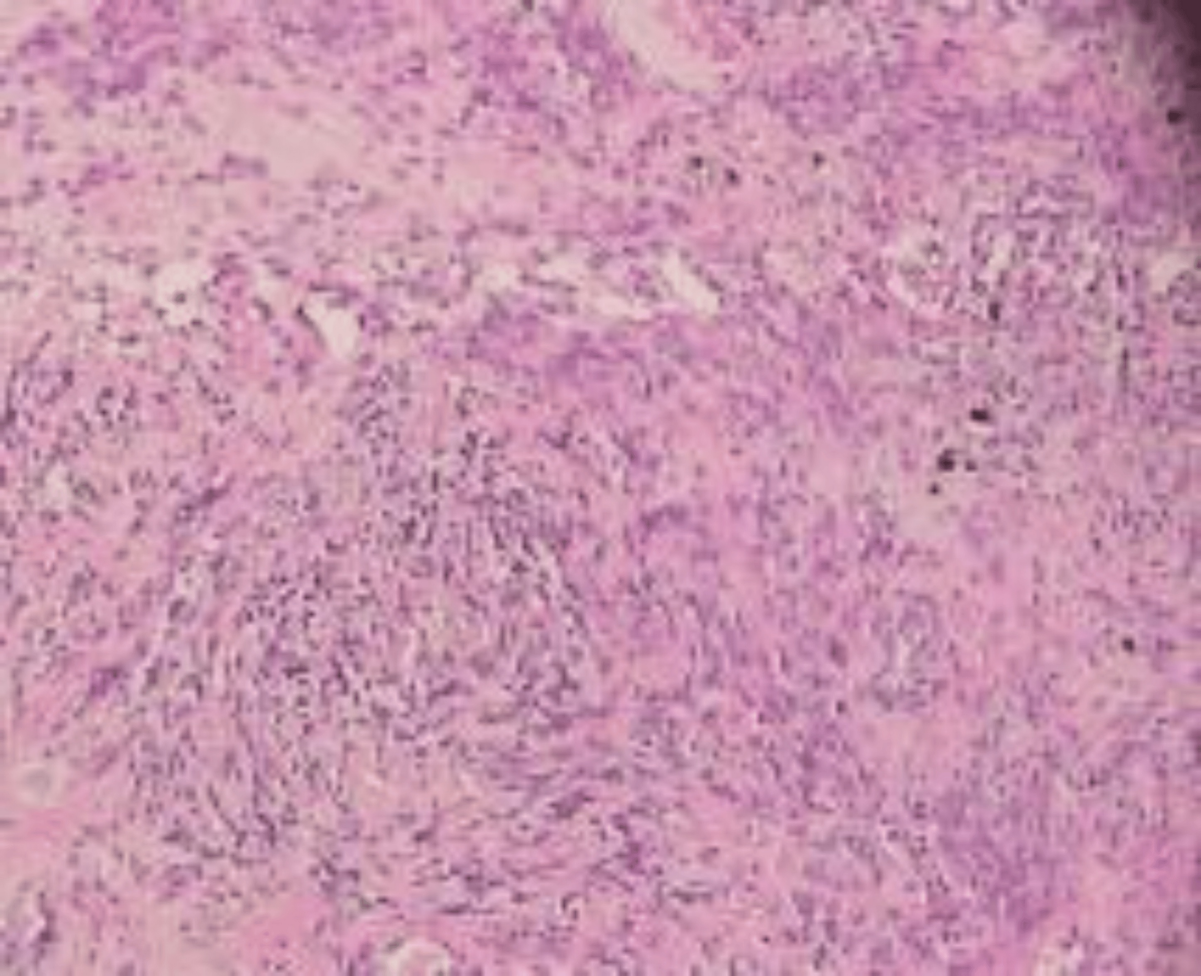
Ovarian perivascular epithelioid cell tumor (H&E stain, ×100 magnification). The photomicrograph demonstrates nests and fascicles of epithelioid to spindle-shaped cells with clear to lightly eosinophilic cytoplasm arranged around a rich capillary network. The nuclei are round to oval with mild pleomorphism. The background stroma shows delicate vascularity without significant necrosis in this field. On further immunohistochemistry, tumor cells were positive for smooth muscle actin (SMA), supporting the diagnosis of PEComa, which typically exhibits both melanocytic and smooth muscle markers.

She subsequently underwent a left salpingo-oophorectomy, excision of a left pelvic wall mass, and infracolic omentectomy. Histopathological examination (Figures 4-6) confirmed primary malignant ovarian PEComa, with metastatic deposits identified in the omentum and left pelvic sidewall. There were no reported intraoperative and immediate postoperative maternal and fetal adverse events. 

The case was re-discussed in the MDT. Given the patient’s early pregnancy, a decision was made to defer systemic targeted therapy until after delivery. She remained disease-free, clinically and radiologically, at her three-month post-delivery follow-up.

## Discussion

Ovarian PEComas have been reported across a broad age range, from 32 to 60 years, with no clear correlation between age and tumor behavior. To our knowledge, our patient, at 31 years old, is the youngest reported case to date. Similar early-onset cases include the 32-year-old described by Sheikhhasani et al. [25], who presented with advanced disease and poor outcome, and the 33-year-old reported by Lee et al. [15], who had a small, TFE3-rearranged tumor of uncertain malignant potential managed successfully with surgery alone. In contrast, older patients such as the 54- to 60-year-olds in the reports by Westaby et al. [26] and Gadducci et al. [22] often exhibited large, invasive tumors with recurrence. Interestingly, both of Schoolmeester’s cases (age 49) had more indolent features and favorable outcomes despite differing lateralities and borders. This spectrum highlights that while PEComas may occur at any adult age, their biological behavior is variable and not age-dependent, ranging from indolent to aggressive, even in young women, as demonstrated by our 31-year-old patient.

In reported cases of ovarian PEComa, the right adnexa appear slightly more commonly involved, as seen in cases by Gadducci et al. [22], Sharma et al. [24], Sheikhhasani et al. [25], Lee et al. [15], and Schoolmeester et al. [4]. However, left-sided tumors have also been documented, including the large malignant PEComa in Westaby et al.’s case [26] and Schoolmeester et al. [4], both involving the left ovary. Our case, involving the left adnexa in a 31-year-old patient, adds to the growing subset of left-sided ovarian PEComas. The available data suggest that PEComas can arise in either ovary, and laterality does not correlate reliably with tumor behavior or prognosis.

The clinical presentation of ovarian PEComas is highly variable, often mimicking other gynecologic or gastrointestinal conditions. The most common complaint is abdominal or pelvic pain, as seen in our 31-year-old patient and in cases reported by Sharma et al. (38 years) [24], Gadducci et al. (60 years) [22], Westaby et al. (54 years) [26], and Schoolmeester (Case 7, 49 years) [4]. Sheikhhasani et al. [25] described a more acute presentation with severe abdominal pain and massive ascites requiring emergency surgery. In contrast, some cases were incidentally discovered during imaging or surgery for unrelated indications, such as the 33-year-old in Lee’s report [15] and Schoolmeester’s Case 10 [4]. These findings emphasize that PEComas may range from asymptomatic and incidental to highly symptomatic masses with systemic involvement. Our patient's presentation with lower abdominal pain falls within the symptomatic spectrum, reinforcing the need for a high index of suspicion, particularly in adnexal masses with unusual imaging or histopathologic features.

Surgical management of ovarian PEComa varies widely depending on tumor size, clinical suspicion, and intraoperative findings. Extensive cytoreductive procedures were performed in aggressive or advanced cases, such as total abdominal hysterectomy with bilateral salpingo-oophorectomy (TAH-BSO) and bowel resection in Westaby’s case [26], and en bloc resection including uterus, adnexa, and bowel segments in Gadducci’s case [22]. Similarly, Sheikhhasani’s patient [25] underwent BSO, omentectomy, and lymphadenectomy due to diffuse peritoneal disease. Our patient, with a left-sided mass, also underwent a fertility-sparing procedure, i.e., left salpingo-oophorectomy + infracolic omentectomy and pelvic lymph node dissection, consistent with approaches for potentially malignant ovarian tumors. In contrast, more conservative surgeries such as unilateral salpingo-oophorectomy were performed in cases with benign or uncertain potential tumors, including Lee’s 33-year-old patient (right oophorectomy) [15] and Schoolmeester Case 10 (right SO) [4]. These comparisons reflect that the extent of surgery often correlates with intraoperative assessment, histological suspicion, and the need for optimal debulking in malignant PEComas. Our case indicated that surgical intervention in pregnancy is safe and might be the only option available for PEComa management during pregnancy. No adverse intraoperative or immediate postoperative maternal and fetal events occurred.

Tumor size among reported ovarian PEComas varies significantly, ranging from small incidental findings to large abdominal masses. Lee et al. [15] and Schoolmeester et al. Case 10 [4] reported tumors measuring 2.5 cm and 4.2 cm, respectively, both classified as of uncertain malignant potential. On the other end of the spectrum, malignant cases in Gadducci et al. (32 cm) [22], Westaby et al. (20 cm) [26], Sheikhhasani et al. (20 cm) [25], and Sharma et al. (10 cm) [24] involved bulky tumors with aggressive clinical behavior. In our case, surgery was performed at a peripheral hospital, and the tumor size was not documented. However, the presence of features including pelvic wall deposits and omental involvement strongly suggested a malignant process. This highlights that while tumor size is a useful prognostic marker, particularly with the ≥5 cm cutoff in the Folpe criteria [16], its absence does not preclude malignant potential, especially when supported by other adverse features.

Imaging findings in ovarian PEComas are often non-specific but can provide valuable clues, especially in large or vascular tumors. CT was the most used modality, as seen in Gadducci’s case, which revealed a large (25 cm) heterogeneous mass with mixed solid and cystic components [22]. Similarly, Sheikhhasani’s patient underwent CT, which showed a large pelvic mass with ascites and bilateral lung nodules suggestive of metastases [25]. In Westaby’s case, postoperative CT identified early recurrence in the mesentery and iliac fossa [26]. In contrast, Lee and Schoolmeester’s cases involved small tumors that were either incidental findings or lacked detailed imaging data due to localized disease [15,4]. In our patient, ultrasound and MRI revealed a residual left adnexal mass with heterogeneous enhancement, aligning with imaging patterns of larger, potentially malignant PEComas. These comparisons suggest that while imaging cannot definitively diagnose PEComa, large size, solid-cystic appearance, and hypervascularity should raise suspicion and prompt further histologic evaluation.

Nonsurgical treatment of ovarian PEComas remains individualized due to the rarity and variable behavior of the disease. Most low-risk or localized tumors, such as those reported by Lee et al. [15] and Schoolmeester et al.'s Case 10 [4], were managed with surgery alone, with no adjuvant therapy required. In contrast, systemic therapy was utilized in more aggressive cases. Sheikhhasani’s patient [25] received four cycles of bleomycin, etoposide, and platinum (BEP) chemotherapy due to peritoneal and pulmonary metastases, although recurrence occurred soon after. Westaby’s patient [26], who developed early recurrence after surgery, was treated with the mTOR inhibitor sirolimus, based on the association of PEComas with mTOR pathway activation. However, chemotherapy was not administered in cases like Sharma’s [24] and Gadducci’s [22] despite malignant histology, and both experienced recurrences, highlighting the uncertain benefit of systemic therapy. Our patient's adjuvant therapy was deferred till the completion of pregnancy. These comparisons underscore that while non-surgical treatment may benefit select high-risk cases, its role remains undefined and requires further investigation.

Microscopically, ovarian PEComas consistently exhibit epithelioid to spindle cell morphology with varying degrees of nuclear atypia, mitotic activity, necrosis, and vascular invasion, features that help stratify tumors into benign, uncertain, or malignant categories. In our case, the tumor showed marked nuclear pleomorphism, necrosis, and mitotic activity >1/50 HPF, aligning with malignant histological criteria. Similar malignant features were observed in Sharma’s case [24], which showed polygonal and spindle cells, necrosis, and a high mitotic index; and in Westaby’s case, with pleomorphic polygonal cells and >20 mitoses/10 HPF [26]. Gadducci’s tumor showed epithelioid and spindle cells in a myxoid stroma with moderate atypia and necrosis [22], while Sheikhhasani reported only mild atypia but widespread metastasis, indicating that histologic appearance may not always predict biological behavior [25]. Conversely, benign or low-risk cases like those of Lee [15] and Schoolmeester Case 10 [4] displayed well-circumscribed tumors with bland nuclei, absence of necrosis, and no mitotic activity. These comparisons highlight the critical role of microscopic assessment in prognostication and therapeutic planning, especially given the unpredictable nature of PEComas. Table 1 summarizes the worrisome features of different classification systems mentioned in the literature that are being used to categorise PEcoma into benign, uncertain malignant potential, and malignant. In this case, it was of malignant category according to all classification systems. 

**Table 1:** Comparison of histologic classification systems for PEComas. * In the original Folpe classification, it is unclear how to consider 1 mitosis per 50 HPF and a PEComa of 5 cm. **Bennet et al. removed 1 mitotic figure per 50 HPF as a worrisome feature compared to Schoolmester et al. HPF: high-power field; PEComas: perivascular epithelioid cell tumors

Data		Modified Folpe [3]	Folpe et al. [16]	Schoolmeester et al. [4]	Bennett et al. [17]
Worrisome features on histology	Size	See below	≥5 cm		
	Growth pattern	Infiltrative		-	
	Nuclear grade	Marked atypia	High nuclear grade and cellularity	High-grade atypia (excluding degenerative atypia)	
	Mitotic rate	See below	>1/50 HPF*	1/50 HPF	>1/50 HPF**
	Necrosis	Present			
	Invasion	Lymphovascular invasion	Vascular invasion	Lymphovascular invasion	
Classification	Benign	≤1 worrisome feature (Size ≥5–<10 cm, infiltrative growth pattern, mitotic rate 2–3 per 50 HPF, no necrosis, lymphovascular invasion)	No worrisome features	Less than 4 worrisome features	-
	Uncertain malignant potential	1 worrisome feature (Size ≥10 cm, isolated marked atypia, mitotic rate ≥4 per 50 HPF, no necrosis, lymph vascular invasion)	One or both of the following features: Nuclear pleomorphism/ multinucleated giant cells only or size >5 cm*	Less than 4 worrisome features	Less than 3 worrisome features
	Malignant	Necrosis or ≥2 worrisome features (Size ≥5 cm, infiltrative growth pattern, mitotic rate >1 per 50 HPF, lymphovascular invasion, marked atypia)	Two or more worrisome features	4 or more worrisome features	3 or more worrisome features

Immunohistochemically, ovarian PEComas characteristically co-express melanocytic and smooth muscle markers, which are key to diagnosis. HMB-45 is the most consistently positive marker across nearly all cases, including ours, and was also reported in Gadducci [22], Sharma [24], Sheikhhasani [25], Westaby [26], Lee [15], and both Schoolmeester cases [4]. Melan-A positivity was variably seen in those by Sheikhhasani [25], Sharma [24], and Schoolmeester [4], while TFE3 nuclear positivity was a distinguishing feature in Lee’s case [15], suggestive of a molecularly distinct subset. Smooth muscle markers such as SMA, desmin, and h-caldesmon were expressed in most malignant cases, including ours, Westaby’s [26], and Sharma’s [24], supporting the dual myomelanocytic differentiation of these tumors. CD10, S-100, and c-kit showed variable and often focal positivity. Ki-67 proliferation index, where reported, was elevated in aggressive cases (e.g., 25-30% in Sharma’s case) [24]. In contrast, benign or uncertain cases such as Schoolmeester Case 10 [4] demonstrated strong melanocytic marker expression but lacked features of high proliferation or aggressive behavior. Overall, our immunohistochemistry (IHC) profile closely aligns with malignant PEComas, reinforcing the value of comprehensive marker panels in diagnosis and risk stratification.

Surveillance practices for ovarian PEComa vary widely due to the rarity and unpredictable behavior of the disease. In high-risk or malignant cases, close imaging-based follow-up was emphasized. Gadducci et al. recommended CT scans every four months and annual brain imaging due to recurrence at 25 months post-surgery [22], while Westaby's patient had a follow-up CT that detected recurrence within four months [26]. In contrast, long-term disease-free survivors such as Schoolmeester Case 7 were monitored over six years with no recurrence, though specific follow-up protocols were not detailed [4]. Similarly, Schoolmeester Case 10 had eight months of uneventful surveillance, and Lee’s patient did not have long-term follow-up data reported [15]. Surveillance for our case included routine pelvic MRI and clinical evaluation at three months post delivery, which is appropriate for malignant PEComa, given the risk of delayed recurrence. These comparisons underscore the importance of individualized, risk-adapted follow-up strategies, with more intensive surveillance warranted in cases with malignant features.

Recurrence and survival outcomes in ovarian PEComas are closely linked to the tumor’s malignant potential and extent of disease at diagnosis. Malignant cases tend to show early or delayed recurrence. Gadducci’s patient experienced local recurrence at 25 months and required reoperation, though she remained disease-free 11 months after resection [22]. Westaby’s case recurred within four months postoperatively and was managed with sirolimus, highlighting the potential for rapid relapse in aggressive variants [26]. Similarly, Sheikhhasani’s patient recurred soon after four cycles of BEP chemotherapy and eventually died of disease progression [25]. In contrast, no recurrence was noted in cases with benign or uncertain potential, such as Schoolmeester Case 7 (disease-free at six years), Case 10 (eight months, no evidence of disease (NED)), and Lee’s patient, though long-term follow-up was limited in the latter two [4,15]. Our patient, managed with fertility-sparing surgery, showed no recurrence at six months post-surgery, contributing to the subset of early disease-free survivors. These comparisons emphasize that recurrence is most common in malignant tumors and may occur despite adequate surgical resection, underscoring the importance of individualized surveillance and further research into prognostic markers to better guide management and improve outcomes. Table 2 summarizes the role of chemotherapy and targeted therapies in ovarian PEComas. 

**Table 2:** Chemotherapy and targeted therapy evidence in ovarian PEComa. ALK: anaplastic lymphoma kinase; CREATE: Clinical Trial of EORTC (European Organisation for Research and Treatment of Cancer) testing crizotinib in rare tumors; MET: mesenchymal-epithelial transition factor; mTOR: mammalian target of rapamycin; TFE3: transcription factor E3; TSC: tuberous sclerosis complex; PEComas: perivascular epithelioid cell tumors

Class / Regimen	Drugs	Rationale / Target	Evidence in Ovarian Perivascular Epithelioid Cell Tumor	Evidence in Other Gynecologic Perivascular Epithelioid Cell Tumors
Conventional chemotherapy	Doxorubicin, Ifosfamide, Dacarbazine; also Vincristine, Cyclophosphamide, Irinotecan, Paclitaxel	Cytotoxic regimens used for soft tissue sarcoma	One malignant adnexal tumor progressed rapidly, and the patient died four months after surgery [21]	– Pediatric uterine tumor treated with vincristine, ifosfamide, and doxorubicin plus radiotherapy remained disease-free for 1.5 years [27] – Uterine tumor with nodal metastasis treated with neoadjuvant chemotherapy, surgery, and adjuvant chemotherapy was disease-free at 8 months [28] – Vaginal tumor recurred after chemotherapy [29]
Mammalian target of rapamycin inhibitors	Sirolimus, Everolimus, Temsirolimus	Target activation of the mammalian target of rapamycin pathway due to loss of tuberous sclerosis complex genes 1 and 2	No ovarian-specific reports, but the rationale applies when molecular alterations are present [30]	– Sirolimus induced temporary regression of lung metastases [31] – Temsirolimus achieved one complete metabolic response, but another case progressed at 22 weeks [32] – Cytoreductive surgery followed by mammalian target of rapamycin inhibitor led to complete response in 2 out of 3 advanced uterine cases [33]
MET/ALK inhibitor (transcription factor E3 rearranged subset)	Crizotinib	Target therapy for transcription factor E3 rearranged tumors, which are less responsive to mammalian target of rapamycin inhibition	One ovarian perivascular epithelioid cell tumor with transcription factor E3 rearrangement has been reported [15]	Transcription factor E3 rearranged tumors are suggested to benefit from crizotinib; activity shown in alveolar soft part sarcoma with transcription factor E3 rearrangement in the CREATE trial [34,35]

A comprehensive review of previously reported ovarian PEComa cases, along with the present case, is presented in Tables 3-5.

**Table 3:** Clinical presentation of ovarian PEComas. h/o: history of; TSC: tuberous sclerosis complex; AML: angiomyolipoma; SOB: shortness of breath; GI: gastrointestinal; MDT: multidisciplinary team; PEComas: perivascular epithelioid cell tumors

Author/Year	Age (years)	Presentation	Presentation Type	Laterality	Tumor Size
Benign					
Anderson et al., 2002 [19]	39	Ovarian cystic mass, h/o TSC & bilateral renal AML	Elective (incidental mass)	Unilateral (ovary, side not specified)	4.5 cm
Rampisela et al., 2016 [20]	43	Irregular periods, adnexal cyst, leiomyomas	Elective (irregular menses, adnexal cyst)	Unilateral (ovary, side not specified)	3.3 × 2.5 cm
Uncertain malignant potential					
Lee & Choi, 2012 [15]	33	Well-circumscribed ovarian solid mass	Elective (solid ovarian mass)	Unilateral (ovary, side not specified)	2.5 × 2 cm
Schoolmeester et al., 2014 (Case 7) [4]	49	Pelvic pain	Elective (pelvic mass workup)	Left adnexa	Not specified
Malignant					
Gadducci et al., 2021 [22]	60	Abdominal pain, large pelvic mass (32 × 28 × 17 cm)	Elective (abdominal pain, large mass)	Unilateral (left ovary)	32 cm
Han et al., 2016 [23]	48	Abdominal discomfort, bilateral ovarian masses, pulmonary metastasis	Elective (abdominal discomfort, metastatic disease)	Bilateral ovaries	Large bilateral masses
Acar & Karaevli, 2024 [18]	46	Melena, abdominal mass invading the transverse colon	Elective (GI bleeding, abdominal mass)	Unilateral (right ovary invading colon)	21 × 14 × 10 cm
Ramaiah et al., 2009 [21]	63	Adnexal mass, shortness of breath, anemia	Elective (adnexal mass, anemia, SOB)	Unilateral (ovary + fallopian tube)	15 × 10 × 9 cm
Sharma et al., 2025 [24]	38	Abdominal pain, fever, right ovarian mass	Emergency (abdominal pain, fever)	Unilateral (right ovary)	10 cm
Sheikhhasani et al., 2022 [25]	32	Acute abdomen, adnexal tumor with ascites, peritoneal and lung nodules	Emergency (acute abdomen, large adnexal mass)	Unilateral (right ovary)	20 × 12 cm
Westaby et al., 2016 [26]	54	Abdominal pain, left ovarian mass with bowel involvement	Elective (abdominal pain, mass found)	Unilateral (left ovary)	20 × 18 × 10 cm
Schoolmeester et al., 2014 (Case 10) [4]	49 (h/o TSC)	Right adnexal mass	Elective (pelvic mass workup)	Right adnexa	4.2 cm
Iqra Yasin et al., 2025 (Current case)	31	Married, para 1, recurrent miscarriages; adnexal mass excised outside, residual mass on imaging	Elective (mass excised, MDT decision)	Unilateral (left ovary)	Residual adnexal mass (size not specified)

**Table 4:** Pathological and investigative findings of ovarian PEComas. AML: angiomyolipoma; CA-125: cancer antigen 125; CK: cytokeratin; CT: computed tomography; EMA: epithelial membrane antigen; ER: estrogen receptor; H-caldesmon: heavy caldesmon; HMB-45: human melanoma black-45; HPF: high-power field; IHC: immunohistochemistry; Ki-67: proliferation index marker; LAM: lymphangioleiomyomatosis; LVSI: lymphovascular space invasion; MRI: magnetic resonance imaging; mets: metastases; PR: progesterone receptor; S100: s100 protein; SMA: smooth muscle actin; TFE3: transcription factor e3; TSC: tuberous sclerosis complex; USG: ultrasonography; Vimentin: intermediate filament of mesenchymal cells

Author/Year	Histology	IHC	Tumor Markers	Associated Conditions	Genetics	Imaging	Folpe/Modified Criteria
Benign							
Anderson et al., 2002 [19]	Epithelioid cells, smooth muscle, thick-walled vessels, adipose tissue	HMB-45+, Melan-A+, SMA+, negative for S100, Desmin, CK	Not reported	TSC + bilateral renal AML	Not reported	Cystic ovarian mass	Benign features
Rampisela et al., 2016 [20]	Epithelioid cells with rhabdoid inclusions	SMA+, Desmin+, HMB-45 (focal), ER/PR+, TFE3 weak	Not reported	Leiomyomas	Weak TFE3	Adnexal cyst	Benign
Uncertain malignant potential							
Lee & Choi, 2012 [15]	Pure epithelioid cells, clear cytoplasm, pigments	HMB-45+, TFE3 strong+, SMA−, Desmin−	Not reported	None	TFE3 rearrangement	Well-circumscribed solid ovarian mass	Uncertain
Schoolmeester et al., 2014 (Case 7) [4]	Hypercellular, pushing border, no necrosis, no LVSI, focal hyalinization	HMB-45+, Melan-A+, SMA+, Desmin+	Not reported	None	Not reported	Not specified	Non-malignant morphology
Malignant							
Gadducci et al., 2021 [22]	Clear/eosinophilic epithelioid & spindle cells, myxoid stroma, necrosis	HMB-45+, H-caldesmon+, Desmin+, actin+, TFE3+, ER/PR weak	Not reported	None	TFE3+, ER/PR weak	Large heterogeneous pelvic mass	Malignant
Han et al., 2016 [23]	Solid/cystic multilocular with hemorrhage, encasing ovarian vessels	Melan-A+, HMB-45+, SMA+	Not reported	Lung metastasis	Not reported	Bilateral ovarian masses + lung mets	Malignant
Acar & Karaevli, 2024 [18]	High nuclear grade, necrosis, mitoses, invasion	HMB-45+, Melan-A+, SMA+	Not reported	None	Not reported	GI bleeding, colon invasion mass	Malignant (necrosis, high grade)
Ramaiah et al., 2009 [21]	Sclerosing PEComa with high-grade malignant transformation	HMB-45+, SMA+, Desmin+	Not reported	None	Not reported	Adnexal solid-cystic tumor	Malignant
Sharma et al., 2025 [24]	Polygonal/spindle cells, pleomorphism, necrosis, hemorrhage	EMA+, Vimentin+, Ki67 25–30%, HMB-45+, SMA+	Not reported	None	Not reported	Right ovarian solid mass with necrosis	Malignant
Sheikhhasani et al., 2022 [25]	Epithelioid nests, mild atypia, hemorrhagic areas	HMB-45+, Melan-A+, SMA+, Desmin+	CA-125 elevated (557 U/mL)	None	Not reported	USG: hypovascular solid tumor; CT: ascites + peritoneal mets + lung nodules	Malignant
Westaby et al., 2016 [25]	High-grade pleomorphic epithelioid cells, necrosis, vascular invasion, >20 mitoses/10 HPF	SMA+, H-caldesmon+, focal HMB45+, Melan-A+, S100+, CD34+, negative EMA	Not reported	None	Not reported	Large necrotic ovarian mass + bowel involvement	Malignant (necrosis, >20 mitoses, LVSI)
Schoolmeester et al., 2014 (Case 10) [4]	Hypercellular, infiltrative border, extensive necrosis (~50%), lymphovascular invasion	HMB-45+, Melan-A+, SMA+, Desmin+	Not reported	TSC, renal AML, pulmonary LAM	Not reported	Right adnexal mass	Malignant (necrosis, infiltrative, LVSI)
Iqra Yasin et al., 2025 (Current case)	Primary ovarian PEComa with metastatic deposits in the omentum and pelvic wall	HMB-45+, SMA+	Not reported	None	Not reported	Residual adnexal mass on USG/MRI	Malignant (metastatic deposits)

**Table 5:** Treatment and outcomes of ovarian PEComas. AML: angiomyolipoma; BSO: bilateral salpingo-oophorectomy; CT: computed tomography; LAM: lymphangioleiomyomatosis; mTOR: mammalian target of rapamycin; NED: no evidence of disease; NOS: not otherwise specified; PEComa: perivascular epithelioid cell tumor; SO: salpingo-oophorectomy; TAH: total abdominal hysterectomy

Author/Year	Surgical Intervention	Fertility/Surgery intent	Adjuvant/Targeted Therapy	Metastasis	Outcome/Follow-up	Follow-up duration
Benign						
Anderson et al., 2002 [19]	Oophorectomy	Radical (oophorectomy)	None reported	None	First ovarian AML reported; benign course	Not reported
Rampisela et al., 2016 [20]	Cystectomy + myomectomy	Fertility-sparing (cystectomy)	None; benign course	None	Benign, no recurrence after 7 years	7 years
Uncertain malignant potential						
Lee & Choi, 2012 [15]	Oophorectomy	Likely fertility-sparing	None	None	First isolated ovarian PEComa NOS	Not reported
Schoolmeester et al., 2014 (Case 7) [4]	Hysterectomy + BSO	Radical (Hysterectomy + BSO)	None	None	Alive, NED at 6 years	6 years
Malignant						
Gadducci et al., 2021 [22]	TAH + BSO + omentectomy + bowel resection	Radical	Recurrence managed surgically; no chemo/radio reported	Recurrence (pelvis)	Recurrence at 25 months, free of disease after resection	25 months
Han et al., 2016 [23]	Bilateral oophorectomy + debulking	Radical debulking	No specific chemo/targeted therapy reported	Lung metastasis	Metastatic disease (lungs)	Not reported
Acar & Karaevli, 2024 [18]	Mass excision + segmental colectomy	Radical	None; follow-up only	None	No recurrence/metastasis at 6 months	6 months
Ramaiah et al., 2009 [21]	TAH + BSO + omentectomy	Radical (TAH + BSO)	No effective therapy; rapid metastasis, no targeted/chemo reported	Lung + liver metastasis	Died of metastatic disease (lungs, liver) within 4 months	4 months (died)
Sharma et al., 2025 [24]	Right salpingo-oophorectomy + omentectomy	Radical (SO + omentectomy)	No adjuvant therapy reported	Omental metastasis	Metastatic deposits in the omentum	Not specified
Sheikhhasani et al., 2022 [25]	Bilateral salpingo-oophorectomy + lymphadenectomy + omentectomy	Radical (BSO + lymphadenectomy + omentectomy)	None reported	Peritoneum + lung nodules	Widespread peritoneal and lung involvement at presentation	Not specified
Westaby et al., 2016 [26]	TAH + BSO + small bowel resection	Radical (TAH + BSO + bowel resection)	Started on sirolimus (mTOR inhibitor), palliative intent	Mesenteric metastasis	Recurrence within 4 months, died after progression despite sirolimus	4 months (recurrence, died)
Schoolmeester et al., 2014 (Case 10) [4]	Right salpingo-oophorectomy	Fertility-sparing (SO)	None	Renal AML + pulmonary LAM (associated lesions, not metastasis)	Alive, NED at 8 months; CT showed renal AML + pulmonary LAM	8 months
Iqra Yasin et al., 2025 (Current case)	Fertility-sparing staging: left salpingo-oophorectomy, pelvic wall mass excision, infracolic omentectomy	Fertility-sparing (SO + omentectomy + pelvic wall mass excision)	Targeted therapy (planned after pregnancy, mTOR inhibitor)	Omentum + pelvic wall	Planned targeted therapy (mTOR inhibitor) after pregnancy; follow-up ongoing	Ongoing

This compilation underscores the rarity of this entity and emphasizes the differences in clinical behavior between benign, uncertain malignant potential, and malignant tumors. To our knowledge, the most significant strength of this case report is that it represents the first documented case of ovarian PEComa diagnosed during pregnancy, adding novel insight into the behavior, diagnosis, and management of this rare tumor in a uniquely challenging clinical context. It highlights the feasibility of fertility-sparing surgery in a young patient with a potentially malignant tumor while preserving obstetric outcomes. The case also offers a detailed comparison with existing literature, emphasizing key clinical, histopathological, and immunohistochemical features, and contributes to the limited pool of early-stage, non-recurrent PEComas managed without adjuvant therapy. The primary limitations include the lack of tumor size documentation, as surgery was performed at a peripheral hospital. Additionally, molecular analysis, such as TFE3 gene rearrangement testing, was not conducted, which could have provided further insight into tumor subtype and behavior. The short follow-up period also limits the ability to assess long-term recurrence risk and pregnancy outcomes beyond delivery.

Given the rarity of PEComas in general and the complete absence of prior cases during this case, it highlights the need for awareness of PEComa as a differential diagnosis in adnexal masses during pregnancy. Future studies should focus on compiling such rare presentations through case registries, encouraging the use of molecular profiling (e.g., TFE3 and mTOR mutations) to guide prognosis and potential targeted therapy. Additionally, long-term follow-up will be essential to assess both oncologic and reproductive outcomes, especially in women undergoing conservative surgery during pregnancy.

## Conclusions

To our knowledge, this case represents the first reported ovarian PEComa diagnosed during pregnancy, adding a unique perspective to the limited literature on this rare tumor. Managed successfully with fertility-sparing surgery, the patient showed classic PEComa features and remains recurrence-free on short-term follow-up.

Given the unpredictable behavior of PEComas, especially in young patients, this case underscores the importance of individualized management, long-term surveillance, and the need for further research into molecular profiling and treatment strategies, particularly in pregnancy-associated presentations.
